# How were pregnant women included in emergency COVID-19 vaccine delivery in The Gambia? A qualitative study of perspectives of healthcare workers and policy influencers

**DOI:** 10.1136/bmjopen-2026-118873

**Published:** 2026-08-20

**Authors:** Oluwatosin Nkereuwem, Alpha Omar Jallow, Alero Ogbebor, James Owolabi, Musa Marena, Sidat Fofanna, Joanna Busza, Uduak Okomo, Beate Kampmann

**Affiliations:** 1 Vaccines and Immunity Theme, Medical Research Council Unit The Gambia at the London School of Hygiene and Tropical Medicine, Fajara, Gambia; 2 Reproductive, Maternal, Newborn Child and Adolescent Health Programme (RMNCAH), Ministry of Health, Kanifing, Gambia; 3 Expanded Programme on Immunization, Ministry of Health, Kotu, Gambia; 4 London School of Hygiene and Tropical Medicine Faculty of Public Health and Policy, London, UK; 5 Medical Research Council Unit The Gambia at the London School of Hygiene and Tropical Medicine, Fajara, Gambia; 6 London School of Hygiene and Tropical Medicine Centre for Maternal Adolescent Reproductive and Child Health, London, UK; 7 Charité Centre for Global Health and Institute for International Health, Charite - Universitatsmedizin Berlin, Berlin, Germany

**Keywords:** COVID-19, Disease Outbreaks, Pregnancy

## Abstract

**Abstract:**

**Objectives:**

Pregnant women are at increased risk of severe illness and adverse pregnancy outcomes during infectious disease outbreaks, yet they are frequently excluded from early emergency vaccine responses. During the COVID-19 pandemic, evolving pregnancy-specific safety evidence contributed to delayed and inconsistent guidance, complicating integration of vaccination into routine maternal health services. This study examined how pregnant women were included in The Gambia’s COVID-19 vaccine rollout and identified priorities to strengthen pregnancy-inclusive preparedness for future emergencies.

**Design:**

We conducted a qualitative exploratory study in The Gambia using semistructured key informant interviews with healthcare workers and policy influencers followed by facilitator-guided group discussions during a participatory workshop. Data were analysed using a hybrid deductive–inductive framework synthesis approach guided by the Consolidated Framework for Implementation Research (CFIR).

**Setting:**

The Gambia, within the Ministry of Health facilities and a national workshop venue. Data were collected between September 2023 and November 2024, following national expansion of COVID-19 vaccine eligibility to pregnant women in The Gambia.

**Participants:**

15 key informants (healthcare workers and policy influencers involved in maternal health, immunisation and vaccine policy) and 29 participants in a subsequent participatory workshop.

**Results:**

Across CFIR domains, implementation was shaped by evolving safety and eligibility guidance, which reduced confidence in counselling and contributed to hesitancy. Outer-setting influences included misinformation and social influence within households and communities, alongside trust in ministry of health actors and endorsement by religious and traditional leaders. Inner-setting constraints included fragmented delivery pathways not integrated into antenatal care, transport and cold-chain challenges, and weak documentation and adverse event reporting that limited visible safety reassurance. At the individual level, healthcare workers were trusted but constrained by staffing pressures, long waiting times and limited access to timely information, while visible role-modelling supported acceptance. Suggested strategies included early training, cross-cadre coordination, strengthened logistics and data systems, improved safety monitoring and communication, and investment in workforce retention and sustainable financing.

**Conclusions:**

Pregnancy-inclusive emergency vaccination requires coordinated action across guidance, community engagement, service organisation, workforce readiness and safety monitoring. Embedding these determinants within routine preparedness, rather than relying on ad hoc emergency responses, can improve equity and effectiveness globally.

**STRENGTHS AND LIMITATIONS OF THIS STUDY**

- This study combined semistructured key informant interviews with a facilitator-guided participatory workshop, enabling triangulation of individual and group-level qualitative accounts.
- Data were analysed using a hybrid deductive–inductive framework synthesis approach guided by the Consolidated Framework for Implementation Research (CFIR), supporting systematic yet context-sensitive interpretation.
- Purposive sampling captured perspectives from healthcare workers and policy influencers across facility, regional and national levels, but did not include pregnant women themselves.
- The retrospective design relied on participant recall of experiences during the COVID-19 rollout, which may be subject to recall bias.
- Findings are context-specific to The Gambia and may have limited transferability, although themes are consistent with evidence from other low- and middle-income country settings.

## Introduction

 Pregnant women are among the most vulnerable groups during infectious disease outbreaks. They face heightened risks of severe illness, adverse pregnancy outcomes and, in some cases, vertical transmission to the infant.^1–3^ Physiological changes in pregnancy, together with altered immune responses, increase susceptibility to severe infection and complicate clinical management during public health emergencies.^4^ Including pregnant women in emergency vaccination programmes is therefore both an equity imperative and a public health necessity, particularly in settings where maternal and neonatal morbidity and mortality remain high and access to timely care is constrained.^3 5 6^ However, the successful implementation of such programmes depends on a complex interplay of health system determinants that are often poorly characterised in low-resource settings.^7^

Globally, emergency vaccination responses have often overlooked pregnant women, especially in low- and middle-income countries (LMICs).^3 5^ During the COVID-19 pandemic, exclusion from early clinical trials generated critical evidence gaps. This contributed to evolving and sometimes inconsistent national guidance, uncertainty among healthcare workers (HCWs) and vaccine hesitancy among pregnant women.^8 9^ Shifts in guidance complicated risk communication and delayed integration of COVID-19 vaccination into routine maternal health services.^10^ In contrast to established maternal vaccines, which are delivered through routine antenatal care (ANC) with well-characterised safety profiles, COVID-19 vaccines were initially deployed mainly through mass campaigns.^11–13^ In many settings, including The Gambia, campaign-style delivery posed operational and coordination challenges for reaching pregnant women through established ANC pathways.^14–16^

Given the lack of published evidence on The Gambia’s experience with including pregnant women in the COVID-19 vaccine rollout, insights from comparable LMICs point to systemic barriers.^17–19^ These barriers span both demand-side and supply-side determinants, including community perceptions and trust as well as facility-readiness challenges such as limited pharmacovigilance capacity and cold-chain infrastructure.^11 20^ Recent outbreaks in West Africa, including Ebola, Lassa fever and a recent mpox case in The Gambia, highlight the urgency of identifying determinants of implementation readiness rather than relying on impromptu emergency responses.^21–24^

HCWs and policy influencers play a pivotal role in these dynamics. Their experiences shape how guidance is interpreted, communicated and operationalised during emergencies and influence how resource allocation and coordination decisions affect delivery within routine maternal services.^25 26^

Guided by the Consolidated Framework for Implementation Research (CFIR),^27^ this study explores the perspectives of HCWs and policy influencers on the inclusion of pregnant women in The Gambia’s COVID-19 vaccine rollout. Using the CFIR to examine multilevel determinants of implementation, we identify actionable priorities to strengthen pregnancy-inclusive vaccine preparedness in similar LMIC contexts.

## Methods

### Study design and setting

We conducted a qualitative exploratory study in The Gambia using key informant interviews (KIIs) and participatory workshop group discussions with HCWs and policy influencers. The Gambia is a West African country with a population of approximately 2.8 million.^28^ Its health system is organised into three tiers (primary, secondary and tertiary) and is decentralised into six health regions.^29^ Data collection took place between September 2023 and November 2024, corresponding to the period following national expansion of COVID-19 vaccine eligibility to pregnant women in The Gambia.

### Theoretical framework

**Figure 1:**
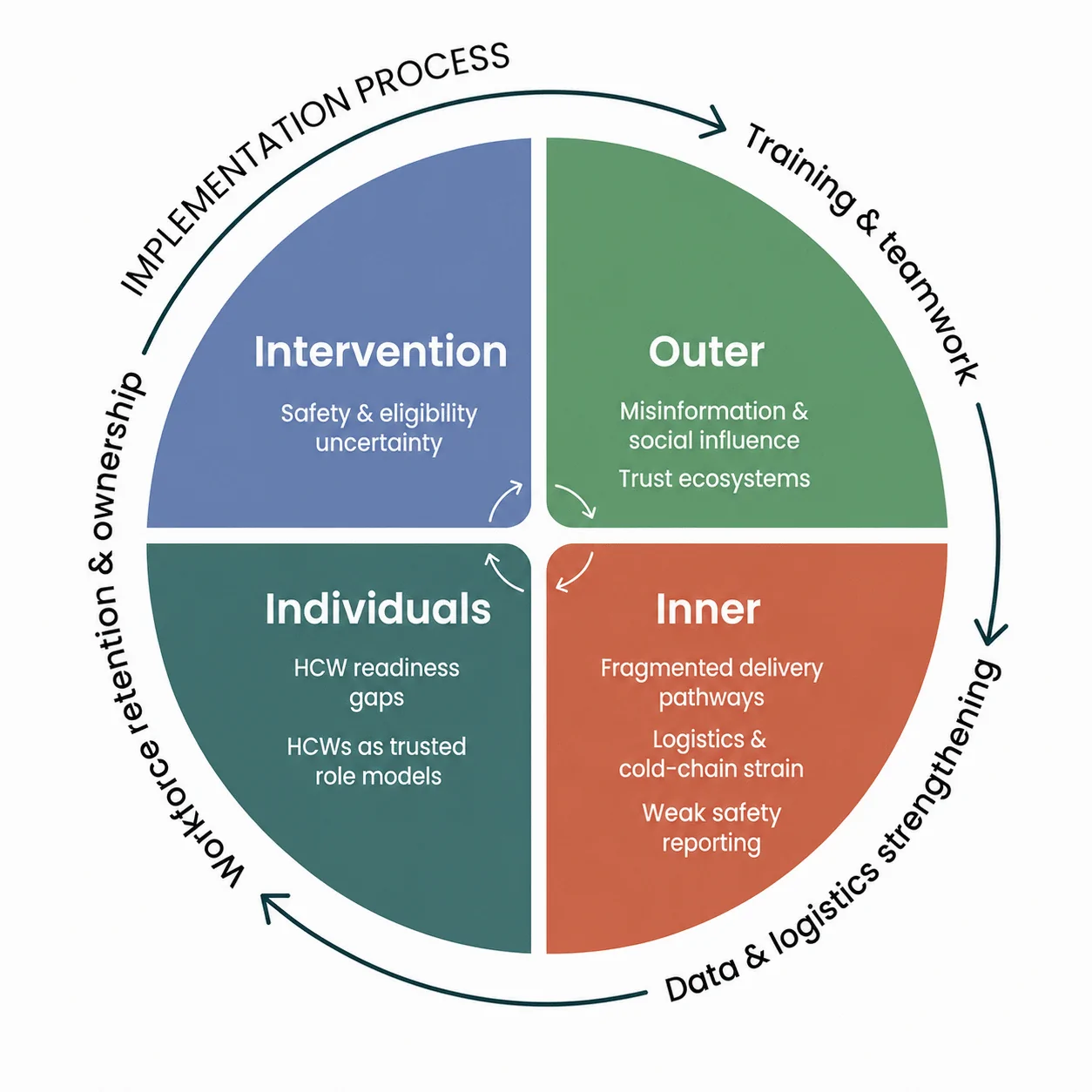
Multilevel determinants of pregnancy-inclusive vaccine implementation in The Gambia (based on CFIR). CFIR, Consolidated Framework for Implementation Research; HCW, healthcare worker.

This study was guided by the CFIR, which enables systematic assessment of multilevel determinants shaping implementation in real-world health systems.^27^ CFIR domains were used to structure data collection and analysis across perceived vaccine characteristics (eg, safety and eligibility clarity), community context (eg, trust and misinformation), organisational readiness (eg, service organisation, logistics and information systems), provider factors (eg, knowledge and confidence) and implementation activities (eg, training and engagement) (figure 1). Using CFIR, we examined how these determinants shaped inclusion of pregnant women during The Gambia’s COVID-19 vaccine rollout and identified priorities to strengthen pregnancy-inclusive preparedness for future public health emergencies.

### Participant sampling

The study was conducted in two sequential phases to capture perspectives from both policy-level actors and frontline providers. In the first phase, we conducted KIIs with purposively selected participants directly responsible for maternal health services, immunisation programmes and vaccine policy. Participants were eligible if they held a decision-making, implementation or service-delivery role in maternal health, immunisation or vaccine policy in The Gambia. These included senior officials from the ministry of health overseeing the Expanded Programme on Immunisation, the Reproductive, Maternal, Newborn, Child and Adolescent Health programme and the Medicines Control Agency. Regional health managers and heads of maternal health service units in major facilities were also included. Participants were selected for their roles in shaping national policy, directing programme implementation and delivering services at national, regional and facility levels. Their perspectives informed the design of the second phase.

In the second phase, data were collected through facilitator-guided group discussions at a participatory workshop. This format enabled broader engagement than individual interviews, helped validate preliminary findings and generated additional insights through collective dialogue across professional groups. Workshop participants comprised purposively selected ministry of health officials, senior hospital administrators, frontline HCWs, public health officers, independent researchers and academic staff. Together, they represented both policy influencers and service providers.

### Data collection

We collected data through semistructured KIIs and facilitated group discussions. Data collection tools were designed to elicit accounts aligned with CFIR domains: perceived vaccine safety and complexity (intervention characteristics), policy guidance and community influence (outer setting), service delivery organisation and information systems (inner setting), HCW knowledge and confidence (characteristics of individuals) and training and engagement strategies (process).

#### Key informant interviews

Semistructured interviews were conducted with purposively selected HCWs and Ministry of Health staff involved in maternal health services, immunisation programmes and COVID-19 vaccine implementation. The interview guide (online supplemental appendix A) explored participants’ roles in vaccine delivery, experiences with policy guidance and challenges encountered during implementation. The guide was refined iteratively as data collection progressed, drawing on insights from earlier interviews.

Interviews were held face-to-face in private settings, at participants’ workplaces, such as the office or other convenient locations within the hospital or office premises. Interviews were conducted in English or, if preferred, in local languages commonly spoken in The Gambia, with interpretation provided by trained research team members. All interviews were audio-recorded with participant consent and lasted approximately 45–90 min. Interviews continued until thematic saturation was reached, defined as the point at which no substantive new issues emerged.

#### Group discussions

Facilitated group discussions took place during a participatory workshop to explore shared experiences and health system dynamics related to maternal COVID-19 vaccination. The workshop was a 1-day event held in a neutral, non-clinical setting to promote open discussion. The group discussion lasted between three and 4 hours. It was led by members of the social science research team and supported by trained research assistants. Each group had at least one senior qualitative researcher and two research assistants to support facilitation, note-taking and logistics.

The workshop began with a plenary session, followed by small-group discussions of about 10 participants each. Topic prompts (online supplemental appendix B), based on initial interview findings, were used to guide the discussion. When participants preferred to speak in local languages, trained members of the research team provided interpretation. Participants interviewed in phase one did not participate in the workshop.

Throughout data collection, the team recorded detailed field notes to document contextual information, key points raised during discussions and non-verbal cues. These notes supported interpretation and informed analysis.

### Data analysis

Data were analysed using a sequential and iterative approach, with data collection and analysis occurring in overlapping cycles. Initial analysis of the KIIs informed the design and focus of the participatory workshops, enabling emerging insights to be explored in greater depth.

To align analysis with CFIR, we used a hybrid deductive–inductive framework synthesis approach. We first coded deductively to organise determinants of the vaccine rollout. We then coded inductively to capture context-specific nuances (eg, the role of traditional community leaders) requiring more granular interpretation. NVivo V.14 supported systematic data management, and themes were refined collaboratively to ensure they remained theoretically grounded and rooted in the Gambian context. Where a CFIR construct within a domain was not represented by a distinct theme in the data, this is stated explicitly in the Results rather than inferred.

### Researchers’ perspectives and reflexivity

The research team comprised nine researchers (five female and four male) with expertise in social science, clinical medicine, public health, implementation science and health policy. The lead author is a social scientist with extensive experience conducting qualitative research on vaccine acceptance and health systems in The Gambia and other LMICs. The coauthors include clinicians involved in maternal and child health programmes, senior ministry of health officials and public health researchers with experience in vaccine delivery and policy analysis.

Given the team’s professional roles, some members had prior familiarity with the Gambian health system and pre-existing professional relationships with participants, which could have influenced interactions and interpretation. To mitigate this, data collection was conducted by trained research assistants who were not ministry of health staff, and interviews and workshop discussions followed standardised guides. The analysis team held regular reflexive discussions to surface assumptions, interrogate alternative explanations and ensure that theme development remained grounded in the dataset. The team’s disciplinary diversity supported triangulation of interpretations across implementation, clinical and policy perspectives, strengthening analytical rigour while prioritising participants’ accounts within the broader health system context.

We reported this study using the Standards for Reporting Qualitative Research (SRQR) to ensure transparency and rigour in qualitative reporting (online supplemental appendix C).^30^

### Patient and public involvement

Patients and/or the public were not involved in the design, or conduct, or reporting, or dissemination plans of this research.

## Results

### Sociodemographic characteristics of participants

A total of 15 participants took part in the KIIs, of whom the majority were male (n=11). Most interview participants had relatively limited professional experience: 10 reported fewer than 5 years of service, 3 reported between 5 and 10 years, and 2 reported more than 10 years. Participants were drawn from across the health system, including facility-level staff (n=9), national-level stakeholders (n=5) and one participant operating at the regional level.

The participatory workshop included 29 participants, with a near-equal gender distribution (14 males and 15 females). Participants represented diverse institutional roles, including facility-based HCWs (n=14), independent researchers (n=11) and stakeholders working at national (n=2) and regional (n=2) levels.

For clarity, findings are presented by CFIR domain, with themes and subthemes summarised in table 1.

**Table 1:** Themes and subthemes that emerged based on the CFIR

Category	Themes	Subthemes
Intervention characteristics	Safety and eligibility uncertainty	Early pregnancy exclusion
		Shifting eligibility guidance
		Thin pregnancy safety evidence
		Residual hesitancy after inclusion
Outer setting	Misinformation and social influence	Social media rumour networks
		Credible antivaccine voices
		Household gatekeeping
		Peer influence and familiar community voices
		Leader endorsement as legitimacy
	Trust ecosystems	Baseline trust in MoH/HCWs
		HCWs as credible messengers
		Trust strained by uncertainty
Inner setting	Fragmented delivery pathways	Campaign delivery over ANC integration
		Parallel pathways, unclear navigation
		Distance and transport barriers
		Queues and clinic bottlenecks
	Logistics and cold-chain strain	Vehicle constraints and competing use
		Unreliable distribution for outreach
		Power instability and generator dependence
		Uneven cold-chain capacity
	Weak safety reporting	Low AEFI reporting culture
		Poor records storage
		Limited data skills/training
		Safety monitoring not visible
Characteristics of individuals	HCW readiness gaps	Information gaps early in rollout
		Limited counselling time
		High workload, low bandwidth
		Low confidence under unclear guidance
	HCWs as trusted role models	Provider credibility as reassurance
		Public vaccination role-modelling
		Trust translated into uptake
Implementation process	Training and teamwork	Pre-rollout sensitisation
		CPD-linked training incentives
		Cross-cadre coordination
	Data and logistics strengthening	Cold-chain and logistics upkeep
		Data quality for planning
		Surveillance and reporting reinforcement
	Workforce retention and ownership	Retention and staffing stability
		Motivation and working conditions
		Community ownership structures
		Domestic financing and sustainability

AEFI, Adverse Event Following Immunisation; ANC, antenatal care; CFIR, Consolidated Framework for Implementation Research; CPD, continuing professional development; HCWs, healthcare workers; MoH, ministry of health.

### CFIR domain 1: intervention characteristics

#### Safety and eligibility uncertainty

Uncertainty surrounding vaccine eligibility and safety for pregnant women emerged as a central challenge. Participants explained that pregnant women were initially excluded from COVID-19 vaccination, and their later inclusion was accompanied by evolving and inconsistently communicated guidance. This shifting eligibility context contributed to uncertainty at the point of care and shaped perceived risk among both providers and pregnant women.

We know that Gambians trust the ministry… but we have seen a bit of mistrust along the way, based on recent events. (KII002)

Health workers themselves described initial uncertainty about vaccine safety, reflecting delayed availability of pregnancy-specific safety data and unclear eligibility criteria. Confidence in recommending and administering vaccines to pregnant women developed only after safety data became available and consensus was reached among national experts, often informed by international guidance.

We were not sure about the safety… Eventually, when the data came out that pregnant women can safely take the vaccines… we were convinced and motivated to give it out. (KII003)

Several participants noted that the phased expansion of eligibility, where priority was initially given to older adults and those with comorbidities, contributed to persistent hesitancy among pregnant women, even after official guidance changed. Participants suggested that early exclusion created lasting doubt that was not fully reversed by later policy updates.

When the vaccination also came initially, the age cohort was the elderly, the sick… then it came to the young, then the pregnant women… So even when pregnant women were advised to take it, many of them did not take the vaccine. (KII007)

This theme highlights how early uncertainty about safety and eligibility shaped later acceptability and provider confidence. Other CFIR intervention-characteristics constructs, including complexity, relative advantage and adaptability, were not identified as salient themes in our data; participants’ accounts centred overwhelmingly on safety and eligibility uncertainty, and we therefore did not report these constructs as separate themes.

### CFIR domain 2: outer setting

#### Misinformation and social influence

Misinformation was described as a persistent challenge, circulating through informal community networks and social media and often amplified by individuals perceived as credible, including trained healthcare professionals. In this outer-setting environment, misinformation shaped perceptions of vaccine safety and risk, while the same social networks influenced how vaccination decisions were negotiated and acted on.

We have somebody who was a trained nurse, he goes around to talk against vaccination… he has a lot of followers on social media… once he realises that you work with the ministry… he will block you. (KII03)

Vaccination decisions were also described as socially mediated rather than purely individual. Acceptance of COVID-19 vaccination often depended on the approval and involvement of influential family members, particularly husbands and in-laws, who frequently acted as key decision-makers within households and mediated women’s access to health services.

Not only the involvement of men but even in-laws, because some in-laws are decision-makers. What the in-law wants, the husband may not agree, so you have to involve the in-laws. (G3P3)

Beyond the household, peer-to-peer influence within communities was described as important, particularly when mobilisation relied on familiar community members who could communicate in locally credible ways during a period of widespread uncertainty.

During Covid-19, they (healthcare workers) mobilised a team from that same community to go and talk to their people. People believe in the people they know better than new people coming to them. (G1P1)

Religious and traditional leaders were described as influential gatekeepers of vaccine acceptance. Engagement with established authority figures, including *Alkalos* (community leaders), Village Development Committees, Imams and marabouts, was viewed as essential for legitimising vaccination efforts and signalling social approval.

Without visiting the Alkalo, you will fail… once the Alkalo accepts it, the whole community will accept it. (G3P2)

… and even the Imams and the marabouts. These marabouts are very influential. (G3P5)

#### Trust ecosystems

Trust in health system actors was described as a key enabling context for vaccine acceptance. Ministry of health staff were widely regarded as credible and authoritative sources of health information within communities, particularly when communicating directly with pregnant women.

Our people have lots of trust in the health care system. (KII007)

The Ministry of Health workers are the most trusted individuals… when a staff member from the Ministry goes around to talk to them, people tend to trust that information better than others. (G1P3)

Across the outer setting, uptake was shaped by competing community narratives and authority structures, alongside a baseline legitimacy for formal health system actors.

### CFIR domain 3: inner setting

#### Fragmented delivery pathways

Service delivery arrangements constrained vaccine access for pregnant women. COVID-19 vaccination was largely delivered through mass campaigns and outreach strategies rather than being integrated into routine ANC, resulting in parallel delivery pathways that were difficult for women to navigate. Structural barriers, including poor road infrastructure, limited transport options, long travel distances and rainy season constraints, restricted access to health facilities.

Distance becomes a major problem… there are no good road networks that would help taxis get across. (KII002)

Travelling to the facilities could be a challenge… some come from extremely far places… or you come and find the queue is too long. (KII015)

#### Logistics and cold-chain strain

Participants described transport constraints as a routine ‘workaround’ problem: when dedicated vehicles were unavailable, teams sometimes relied on ambulances or personal funds to move vaccines and supplies. However, ambulances were frequently prioritised for emergency referrals, disrupting planned vaccine distribution and making facility availability less predictable.

Sometimes it’s difficult because we use the ambulances when they are on referral services. (KII003)

Others described compensating for gaps in government transport by paying out-of-pocket to support vaccine movement, illustrating how implementation sometimes depended on individual resources rather than routine system support.

Logistic movement is a problem… Sometimes I pay my transportation fare… Getting vehicles for transportation is a problem. (G1P9)

Cold-chain management was another recurring concern, particularly in settings with unreliable electricity. Reliance on generators increased operational costs and raised concerns about maintaining vaccine potency.

The running of the cold room is another challenge because electricity is very inconsistent in this part of the country, and we have to rely on a standby generator that consumes a lot of fuel. (KII003)

Some facilities benefited from solar-powered refrigerators. However, participants noted uneven infrastructure capacity across regions, contributing to geographical variation in service reliability.

I don’t think cold chain is a problem in some places because some refrigerators are solar-powered. (G3P5)

#### Weak safety reporting

Weak documentation and surveillance systems constrained the generation and circulation of credible safety information, thereby undermining confidence in formal vaccine safety monitoring.

We are not reporting, we are not reporting at all… it is very important to write and send reports of such adverse events. (G3P6)

Inadequate storage space and limited training in data management further constrained record-keeping and follow-up, reducing the visibility of vaccine safety processes within routine care.

Record keeping is also one of the systemic problems because some facilities don’t even have space or storage facilities to keep records. (G2P1)

In this context, gaps in safety reporting were seen as limiting the health system’s ability to demonstrate that monitoring was occurring. Taken together, inner-setting constraints affected access, service continuity and the visibility of safety monitoring, each critical for pregnancy-inclusive delivery.

### CFIR domain 4: characteristics of individuals

#### Healthcare worker readiness gaps

Staff shortages were repeatedly described as constraining vaccine delivery. Limited staffing increased workloads, prolonged waiting times and reduced opportunities for counselling, particularly in busy ANCs where women were required to move between multiple service points, intensifying pressure on both providers and patients.

What we usually do is to have clinic health talks… But the information we provide is not enough for pregnant women… many don’t give the information as required. (KII012)

Extended waiting times were described as physically and emotionally exhausting for pregnant women, contributing to frustration and disengagement from services.

… pregnant women are also agitated if they queue for a long time and are tired… You even call their names, and they would be sleeping… they tend to be furious… (KII013)

The cumulative effect of limited staffing and high patient volumes was succinctly captured by another participant:

Yes, we experience long waiting times. Long waiting hours. Limited staff and plenty of people to take care of, then that becomes a challenge because the workload will increase. (G3P4)

Early in the rollout, inadequate preparation and limited access to timely information constrained providers’ confidence in responding to pregnant women’s concerns.

For me, as the health worker, I was not prepared. I did not have enough information, so when people come to me, I tell them I don’t know… the vaccine was kind of rushed… everybody was confused… the acceptance rate was low. (G3P4)

These accounts reflect readiness constraints driven by both limited capacity (time, staffing) and limited confidence in recommending vaccination during evolving guidance.

#### Healthcare workers as trusted role-models

Visible role-modelling by HCWs was described as a particularly persuasive strategy for encouraging vaccine uptake. Participants explained that when frontline staff received the COVID-19 vaccine publicly, it reassured pregnant women and demonstrated confidence in vaccine safety in a way that verbal communication alone could not achieve.

I told the people I would take it in front of you. I took it in front of everybody. This place was full, so some of them saw me. So that is what I used. I took the COVID-19 vaccine in front of everybody, and here I am. (KII013)

The influential factors are health workers leading the way… when the COVID-19 vaccine came, where health workers were at the front to receive this vaccine, we think people were also encouraged to receive the vaccine. (G1P2)

At the individual level, provider readiness constraints co-existed with high credibility, with role-modelling used to strengthen reassurance when safety questions were prominent.

### CFIR domain 5: implementation process

#### Training and teamwork

Forward-looking strategies emphasised strengthening readiness for pregnancy-inclusive vaccination beyond COVID-19. Timely, structured training was viewed as necessary so that HCWs could provide accurate, pregnancy-specific information and address concerns and misinformation.

Healthcare workers have limited knowledge about vaccines like the COVID-19 vaccine… so if a new vaccine is also introduced, I think there should be sensitisation before the vaccine comes. (G3P5)

To enhance uptake and sustainability of training, participants proposed embedding vaccine-related education within formal continuing professional development (CPD) frameworks, thereby aligning preparedness activities with professional incentives.

Our recommendation is to train the healthcare workers… it should be introduced on the continuing professional development (CPD) points so that it will attract people who are going to do the course. (G1P2)

Beyond individual training, participants stressed the importance of inter-cadre collaboration. Siloed roles between public health officers, nurses and other frontline staff were perceived to undermine efficiency and continuity of care, particularly during emergency responses.

It should not be seen as an issue of a particular healthcare cadre, but should be inclusive of all cadres… collaboration between public health officers and nurses is very important. It will ease many of the burdens. (G1P7)

#### Data and logistics strengthening

Infrastructure and data systems were prioritised as critical enablers of effective and sustainable immunisation services. Reliable logistics and cold chain capacity were viewed as essential for maintaining vaccine quality and ensuring uninterrupted service delivery, particularly in resource-constrained settings.

The fueling and the logistics, the cold chain maintenance, is very important… it should be maintained to the last vial. (G1P6)

Robust data systems were similarly prioritised for programme monitoring, safety oversight and accountability. Participants underscored that accurate, timely data underpin both national planning and donor confidence.

The accurate provision of data is important in our system… with correct data, this can help us… donors will depend on this data to help. (G3P4)

#### Workforce retention and ownership

Sustaining a skilled and motivated workforce emerged as a persistent concern. Participants highlighted high attrition rates among trained HCWs, which threatened continuity of services and undermined investments in training and capacity building.

Despite the quality training the schools are producing, staff are not retained… there should be strategies for retaining the staff that are produced. (G1P2)

Motivation was repeatedly described as a determinant of performance and commitment, with poor working conditions and limited incentives contributing to disengagement.

Healthcare workers need motivation… if they are not motivated, they will be discouraged. (G1P6)

In parallel, participants emphasised that workforce strengthening should extend beyond clinical staff to include community engagement actors. Involving men, religious leaders, *Alkalos* and griots (*Kenyelings*) was viewed as a way to build local ownership and reduce the burden on overstretched health workers.

For me, what I will always be emphasising is the involvement of men, the involvement of religious leaders, and making sure communities feel like they own this. (G3P6)

Participants underscored the importance of government leadership and sustainable financing to support workforce stability, cautioning against reliance on short-term external funding.

The government needs to support immunisation programs not only by depending on external partners. (KII002)

Let us have a sustainability plan and try to include it in our current budget… if partners’ funding dwindles, the government can still keep it. (KII003)

### Cross-domain synthesis

Determinants of pregnancy inclusion spanned all CFIR domains and were seen as interconnected but not identical. Safety and eligibility uncertainties (intervention characteristics) affected acceptability; misinformation, household and community decision-making structures and broader trust ecosystems (outer setting) shaped demand and perceptions of legitimacy; fragmented delivery pathways, logistical constraints and weak safety reporting systems (inner setting) limited both access and visible reassurance; and readiness gaps among HCWs (characteristics of individuals) constrained counselling early in rollout. In response, proposed strategies sought to strengthen implementation by investing in training and teamwork, improving data systems and logistics reliability, supporting workforce retention and fostering stronger community ownership.

## Discussion

This study shows how The Gambia’s COVID-19 vaccine rollout tested the health system’s ability to implement pregnancy-inclusive vaccination during an emergency. Using CFIR to interpret perspectives from HCWs and policy influencers, we found that inclusion was shaped by determinants spanning the intervention itself, the community context, organisational readiness and provider preparedness. Adaptive capacities, including trust in health system actors, engagement of community leaders and visible HCW role-modelling, supported uptake despite these constraints. The findings highlight practical priorities for pregnancy-inclusive preparedness that extend beyond vaccine supply to the systems and relationships required for delivery.

### Intervention characteristics: safety and eligibility uncertainty

The initial exclusion and later delayed inclusion of pregnant women during the COVID-19 vaccine rollout in The Gambia reflects a broader pattern observed during public health emergencies, where uncertainty about pregnancy-specific safety data leads to cautious or delayed eligibility decisions.^8 31^ Delays in eligibility have been associated with confusion among providers and reduced confidence among pregnant women, even after guidance changes.^19 32 33^ In this study, evolving eligibility and limited pregnancy-specific evidence early in the rollout led to uncertainty at the point of care, with providers describing difficulty providing clear reassurance when guidance was changing. Evidence from multicountry surveys indicates that vaccine acceptance is strongly shaped by perceived safety and efficacy, underscoring the importance of timely risk communication and clear eligibility guidance during emergency vaccination programmes.^34^

Accounts also suggested that early exclusion had enduring effects on confidence and uptake, reinforcing the point that pregnancy inclusion cannot be treated as a late-stage adjustment. These findings underscore the value of pregnancy-inclusive preparedness frameworks that enable timely adaptation as safety evidence emerges.^35^ Preparedness should include clear decision pathways for pregnancy eligibility, rapid dissemination mechanisms for updated guidance and aligned communication materials that can be deployed promptly as evidence evolves.^36^

### Outer setting: misinformation, social influence and trust ecosystems

Community context strongly shaped acceptability and legitimacy. Misinformation circulating through social media and community networks, sometimes amplified by credible voices, competed with official messaging and reinforced safety concerns. Decisions were also described as socially mediated, with household decision-makers influencing women’s access and religious and traditional leaders shaping community acceptance. Consistent with evidence from emergency vaccination efforts in similar settings, endorsement by community leaders can legitimise vaccination by aligning it with existing authority structures and shared norms.^7 37^ In The Gambia, figures such as *Alkalos*, Imams and *marabouts* have been described as influential, while husbands and in-laws have often mediated women’s access to vaccination.^38^

A notable and somewhat contradictory finding was that a trained nurse was reported to be actively campaigning against vaccination within the community. This suggests that clinical training does not guarantee alignment with public health guidance, and that credible antivaccine voices carrying particular authority can emerge from within the health workforce itself.^39^ Unspoken doubt and hesitancy among HCWs have been documented elsewhere, with implications for trust-building and workforce management, including a need for internal engagement and accountability mechanisms that allow workers’ own doubts to be surfaced before they are expressed publicly against official guidance.^40^

Preparedness should move beyond ad hoc engagement to formalise relationships with trusted leaders, maintain updated engagement plans and pretest messages that can be rapidly deployed during outbreaks. These strategies can reduce delays, support HCWs and strengthen community-level confidence.

### Inner setting: delivery pathways, logistics and safety reporting

Constraints within routine delivery systems further limited pregnancy-inclusive vaccination. Campaign-style delivery that was not integrated into ANC created parallel pathways that were harder for pregnant women to navigate and more difficult for providers to coordinate. Persistent weaknesses in logistics, transport and cold-chain reliability, including inconsistent electricity supply and limited vehicles, mirror broader evidence from LMIC settings that fragile infrastructure undermines reliable delivery and can weaken confidence in vaccination, particularly among pregnant populations.^13 41^

Weak documentation and adverse event reporting reduced the visibility of safety monitoring processes, limiting the health system’s ability to provide credible reassurance during periods of uncertainty. In contexts where pregnancy safety concerns are prominent, the ability to demonstrate that safety monitoring is functioning and to communicate what is being observed becomes especially important.^42^ Strengthening functional, visible pharmacovigilance systems, including systematic adverse-event-following-immunisation reporting and feedback, has similarly been identified as essential for building public confidence in vaccine safety during emergency rollouts targeting vulnerable groups such as pregnant women.^43^

### Characteristics of individuals: HCWs as trusted but constrained implementers

HCWs were central to shaping maternal vaccine acceptance, consistent with evidence that provider recommendation is a strong determinant of uptake. Visible role-modelling by HCWs, including receiving vaccination publicly, was described as an effective way to translate safety assurances into tangible reassurance.^44^ However, trust in HCWs was dependent on institutional support.^45^ Inadequate training, limited risk communication capacity and poor access to timely information left some HCWs feeling underprepared, limiting their ability to counsel confidently and respond to misinformation.

Beyond its immediate reassurance value, role-modelling could be formalised as a structured intervention rather than left to emerge spontaneously. Approaches such as designating trained ‘vaccine champions’ to lead public vaccination events and pair visible demonstration with structured counselling have improved vaccine confidence and uptake in comparable low-resource settings.^46^ Embedding role-modelling within workforce training and communication strategies could help translate this adaptive strength into a scalable preparedness tool.

Workforce preparedness should therefore combine technical content, practical risk communication skills and rapid feedback mechanisms that help providers respond to evolving guidance.^47^ Planning should also consider how service platforms can support counselling at scale, including integration of vaccination into ANC where feasible and provision of additional staffing or task-sharing during periods of high demand.

### Implementation process: strengthening preparedness beyond the emergency

Recommendations converged on strengthening implementation processes before and during outbreaks. Priorities included early training, cross-cadre coordination, stronger logistics and data systems, and addressing workforce retention and motivation.^48^ Calls for government ownership and domestic financing reflect recognition that reliance on external partners may undermine long-term resilience. Embedding preparedness activities into routine health system functions, including periodic training updates, data quality strengthening and standing plans for community engagement, may reduce the need for improvisation during future emergencies.

### Implications for policy and practice

These findings suggest that pregnancy-inclusive vaccination should be proactively embedded within emergency preparedness planning rather than addressed reactively once vaccines are deployed.^38^ Practical priorities include integrating emergency vaccines within ANC pathways where feasible, strengthening workforce training and risk communication in advance of rollouts, investing in robust data and pharmacovigilance systems, and institutionalising engagement with trusted community leaders. Addressing these structural and relational dimensions in parallel can enhance both equity and effectiveness in future emergency responses.

These lessons are especially relevant as vaccines for emerging infections such as Lassa fever and Ebola become increasingly available and are likely to be deployed through campaign-style strategies.^21–23^ Ensuring that pregnant women are included early, with clear guidance and implementation support, will help avoid delayed protection observed during COVID-19 in The Gambia and similar settings.

### Strengths and limitations

This study provides context-specific insights into pregnancy-inclusive emergency vaccination in The Gambia, where published evidence remains limited. Strengths include diverse perspectives spanning HCWs, policy actors, researchers and academics, enabling examination of frontline delivery and system-level decision-making. The qualitative approach enabled exploration of issues such as trust, misinformation, workforce constraints and service organisation that are difficult to capture quantitatively.

Limitations should be considered. The retrospective design may have introduced recall bias. Findings are context-specific, although themes align with evidence from other LMIC settings. The absence of pregnant women’s perspectives limits triangulation with lived experience. Future studies combining provider and policy perspectives with pregnant women’s accounts and routine service data could strengthen understanding of how determinants vary by geography and delivery platform.

### Conclusion

The COVID-19 vaccine rollout in The Gambia exposed weaknesses in eligibility guidance, service integration, safety reporting and workforce capacity, while also revealing adaptive strengths including social trust, engaged community leadership and visible professional role-modelling. These findings highlight the urgency of pregnancy-inclusive preparedness for future public health emergencies. Priorities include timely guidance, integration with ANC where feasible, strengthened risk communication, robust safety monitoring and sustainable financing, alongside structured community engagement. Addressing determinants across CFIR domains in a coordinated way can strengthen pregnancy-inclusive outbreak vaccination in The Gambia and comparable low-resource settings. These findings should be interpreted in light of the study’s context-specific, retrospective qualitative design and the absence of pregnant women’s own perspectives.

## Supplementary material

10.1136/bmjopen-2026-118873 online supplemental file 1

10.1136/bmjopen-2026-118873 online supplemental file 2

10.1136/bmjopen-2026-118873 online supplemental file 3

## Data Availability

Data are available upon reasonable request.
