# Supplementary material for: How were pregnant women included in emergency COVID-19 vaccine delivery in The Gambia? A qualitative study of perspectives of healthcare workers and policy influencers
Source: BMJ Open. 2026 Aug 20;16(8):e118873. doi: 10.1136/bmjopen-2026-118873 (PMC13504901; doi:10.1136/bmjopen-2026-118873)
Supplement: Supplementary file 1 [file bmjopen-2026-118873.reviewer_comments.pdf]

PEER REVIEW HISTORY

BMJ Open publishes all reviews undertaken for accepted manuscripts. Reviewers are asked to complete a checklist review form and are provided with free text boxes to elaborate on their assessment. These free text comments are reproduced below.

ARTICLE DETAILS

Title (Provisional)

How were pregnant women included in emergency COVID-19 vaccine delivery in The Gambia? A qualitative study of perspectives of healthcare workers and policy influencers

Authors

Nkereuwem, Oluwatosin; Jallow, Alpha Omar; Ogbebor, Alero; Owolabi, James; Marena, Musa; Fofanna, Sidat; Busza, Joanna; Okomo, Uduak; Kampmann, Beate

---

VERSION 1 - REVIEW

**Reviewer**	**1**
**Name**	**Harapan, H**
**Affiliation**	**Universitas Syiah Kuala - Kampus Darussalam**
**Date**	**15-Jun-2026**
**COI**	**None**

---

1. The manuscript does not report the data collection period (timeline) in either the abstract or the methods section. Please specify the months and years during which the key informant interviews and the participatory workshop were conducted, and clarify how this timing relates to the COVID-19 pandemic and vaccine rollout phases in The Gambia.

2. The inclusion/exclusion criteria for KII participants are not clearly described. Please provide specific criteria, for instance, minimum experience, geographic representation, and role types, to justify the purposive sampling strategy.

3. The results for CFIR Domain 1 (Intervention characteristics) report only one theme (safety and eligibility uncertainty), while CFIR includes other constructs such as complexity, relative advantage, and adaptability. Please clarify whether these constructs were not identified in the data or explain why they were excluded from analysis. If they were absent, state this explicitly.

4. In Table 1, the fifth domain is labelled as "Implementation process (and Inner Setting readiness)", which creates confusion and inconsistency with the narrative results (which use "Implementation process") and the methods (which list 5 CFIR domains without this

addition). Please remove the parenthetical "(and Inner Setting readiness)" to align with standard CFIR terminology and your own reporting.

5. Consider citing Rosiello et al. (2021; https://pubmed.ncbi.nlm.nih.gov/38450212/) in the Discussion subsection “Intervention characteristics: safety and eligibility uncertainty,” particularly after the discussion on evolving guidance and limited safety evidence. Their study supports the point that vaccine acceptance is closely shaped by perceived safety and efficacy, thereby strengthening the argument for timely risk communication and clear eligibility guidance during emergency vaccination programmes.

6. The finding that a trained nurse actively campaigned against vaccination represents a contradictory and important theme. However, the Discussion does not address this finding. Please discuss why this might occur, whether it is unique to The Gambia or observed elsewhere, and what implications it has for trust-building and workforce management.

7. The Results section highlights healthcare worker role-modelling as a strong adaptive finding, but the Discussion gives it only brief attention compared to barriers. Please expand the Discussion to more fully address the strategic implications of this finding, including how role-modelling can be systematically leveraged in future emergency preparedness.

8. Consider citing Umar et al. (2025; https://doi.org/10.52225/narrax.v3i3.230) in the Discussion section, particularly in the subsection “Inner setting: delivery pathways, logistics, and safety reporting,” after the paragraph discussing weak documentation and adverse event reporting. This citation would strengthen the argument that visible and functional pharmacovigilance systems are essential for building confidence in vaccine safety, especially when vaccinating pregnant women during emergency rollouts.

9. The Conclusion does not mention any study limitations, which may lead readers to overgeneralise the findings. Please add one brief sentence acknowledging key limitations to provide a balanced interpretation.

VERSION 1 - AUTHOR RESPONSE

Reviewer: 1
Dr. H Harapan, Universitas Syiah Kuala - Kampus Darussalam

Reviewer’s comments	Author’s responses
1. The manuscript does not report the data collection period (timeline) in either the abstract or the methods section. Please specify the months and years during which the key informant interviews and the participatory workshop were conducted, and clarify how this timing relates to the COVID-19 pandemic and vaccine rollout phases in The Gambia.	We agree this was an important omission. We have added the data collection period to both the abstract (Setting) and the Methods (Study design and setting), and have related this timing explicitly to the phase of

	the national COVID-19 vaccine rollout.
2. The inclusion/exclusion criteria for KII participants are not clearly described. Please provide specific criteria, for instance, minimum experience, geographic representation, and role types, to justify the purposive sampling strategy.	We have added an explicit statement of eligibility criteria for key informants to the Participant sampling section, specifying the minimum role-related experience required, the professional cadres eligible, and the facility/regional/national representation sought, consistent with the purposive sampling approach already described.
3. The results for CFIR Domain 1 (Intervention characteristics) report only one theme (safety and eligibility uncertainty), while CFIR includes other constructs such as complexity, relative advantage, and adaptability. Please clarify whether these constructs were not identified in the data or explain why they were excluded from analysis. If they were absent, state this explicitly.	We confirm that complexity, relative advantage, and adaptability were considered during deductive coding but did not emerge as distinct, well-supported themes in our dataset; participant accounts within this domain centred overwhelmingly on safety and eligibility uncertainty. We have added an explicit statement to this effect in the Results, and a general clarifying sentence in the Data Analysis section describing how absent constructs are reported.<br>Sentence added at the end of the Domain 1 results subsection: *“Other CFIR intervention-characteristics constructs, including complexity, relative advantage, and adaptability, were not identified as salient themes in our data; participants' accounts centred overwhelmingly on safety and eligibility uncertainty, and we therefore did not report these constructs as separate themes.”*<br>Sentence added to Methods – Data Analysis: *“Where a CFIR construct within a domain was not represented by a distinct theme in the data, this is stated explicitly in the Results rather than inferred.”*
4. In Table 1, the fifth domain is labelled as "Implementation process (and Inner Setting readiness)", which creates confusion and inconsistency with the	We thank the reviewer for identifying this inconsistency. The parenthetical qualifier has been

narrative results (which use "Implementation process") and the methods (which list 5 CFIR domains without this addition). Please remove the parenthetical "(and Inner Setting readiness)" to align with standard CFIR terminology and your own reporting.	removed so that the domain is labelled 'Implementation process' throughout the table, consistent with the Results, Discussion, and Methods.<br>Table 1, final domain row now reads: *Implementation process*.
5. Consider citing Rosiello et al. (2021; https://pubmed.ncbi.nlm.nih.gov/38450212/) in the Discussion subsection "Intervention characteristics: safety and eligibility uncertainty," particularly after the discussion on evolving guidance and limited safety evidence. Their study supports the point that vaccine acceptance is closely shaped by perceived safety and efficacy, thereby strengthening the argument for timely risk communication and clear eligibility guidance during emergency vaccination programmes.	We have added this citation at the suggested location, with a sentence linking the multi-country evidence on perceived safety/efficacy and acceptance to our argument for timely risk communication and clear eligibility guidance. The added sentence reads as follows:<br>*"Evidence from multi-country surveys indicates that vaccine acceptance is strongly shaped by perceived safety and efficacy, underscoring the importance of timely risk communication and clear eligibility guidance during emergency vaccination programmes."*
6. The finding that a trained nurse actively campaigned against vaccination represents a contradictory and important theme. However, the Discussion does not address this finding. Please discuss why this might occur, whether it is unique to The Gambia or observed elsewhere, and what implications it has for trust-building and workforce management.	We agree this finding warranted discussion. We have added a paragraph to the 'Outer setting' discussion subsection addressing why credible anti-vaccine voices can emerge from within the health workforce itself, noting that this is not unique to The Gambia, and drawing out implications for internal trust-building and workforce management. The paragraph reads as follows:<br>*"A notable and somewhat contradictory finding was that a trained nurse was reported to be actively campaigning against vaccination within the community. This suggests that clinical training does not guarantee alignment with public health guidance, and that credible anti-vaccine voices carrying particular authority can emerge from within the health workforce itself.[39] Unspoken doubt*

	and hesitancy among healthcare workers have been documented elsewhere, with implications for trust-building and workforce management, including a need for internal engagement and accountability mechanisms that allow workers' own doubts to be surfaced before they are expressed publicly against official guidance."
7. The Results section highlights healthcare worker role-modelling as a strong adaptive finding, but the Discussion gives it only brief attention compared to barriers. Please expand the Discussion to more fully address the strategic implications of this finding, including how role-modelling can be systematically leveraged in future emergency preparedness.	We have expanded the 'Characteristics of individuals' discussion subsection with a new paragraph on the strategic potential of role-modelling, including concrete suggestions for how it could be formalised within future preparedness planning. It reads as follows:<br>*"Beyond its immediate reassurance value, role-modelling could be formalised as a structured intervention rather than left to emerge spontaneously. Approaches such as designating trained 'vaccine champions' to lead public vaccination events and pair visible demonstration with structured counselling have improved vaccine confidence and uptake in comparable low-resource settings.[46] Embedding role-modelling within workforce training and communication strategies could help translate this adaptive strength into a scalable preparedness tool."*
8. Consider citing Umar et al. (2025; https://doi.org/10.52225/narrax.v3i3.230) in the Discussion section, particularly in the subsection "Inner setting: delivery pathways, logistics, and safety reporting," after the paragraph discussing weak documentation and adverse event reporting. This citation would strengthen the argument that visible and functional pharmacovigilance systems are essential for building confidence in vaccine safety, especially when vaccinating pregnant women during emergency rollouts.	We have added this citation at the suggested location, with a sentence linking it to the importance of functional, visible pharmacovigilance for building confidence in vaccine safety among pregnant women. The sentence reads as follows:<br>*"Strengthening functional, visible pharmacovigilance systems, including systematic adverse-event-following-immunisation*

	reporting and feedback, has similarly been identified as essential for building public confidence in vaccine safety during emergency rollouts targeting vulnerable groups such as pregnant women."
9. The Conclusion does not mention any study limitations, which may lead readers to overgeneralise the findings. Please add one brief sentence acknowledging key limitations to provide a balanced interpretation.	We have added one sentence to the 'Conclusion' acknowledging the study's design-related limitations and the absence of pregnant women's own perspectives. The sentence reads as follows:<br>*"These findings should be interpreted in light of the study's context-specific, retrospective qualitative design and the absence of pregnant women's own perspectives."*

VERSION 2 - REVIEW

**Reviewer**	**1**
**Name**	**Harapan, H**
**Affiliation**	**Universitas Syiah Kuala - Kampus Darussalam**
**Date**	**31-Jul-2026**
**COI**	

I have re-reviewed the revised manuscript and the authors' responses to the previous comments. I am satisfied that all my concerns have been addressed thoroughly and appropriately. The revisions have significantly strengthened the paper. I have no further suggestions or critiques. Therefore, I recommend that this manuscript be accepted for publication.
