# Supplementary material for: How were pregnant women included in emergency COVID-19 vaccine delivery in The Gambia? A qualitative study of perspectives of healthcare workers and policy influencers
Source: BMJ Open. 2026 Aug 20;16(8):e118873. doi: 10.1136/bmjopen-2026-118873 (PMC13504901; doi:10.1136/bmjopen-2026-118873)
Supplement: Supplementary file 2 [file bmjopen-16-8-s001.docx]

# **Key Informant Interviews (KII) Facilitator’s Guide**

**Introduction**

**Good morning,**

**My name is ____________________, and here with me is my (translator/assistant) _____________________. I will be interviewing you today.**

**I want to thank you for consenting to be part of this study and for taking part in this discussion/interview. As described in the study information sheet and informed consent forms, we will record this session and take notes for proper documentation. This session will last for about 30 minutes or more.**

**We promise to keep all information shared here confidential. Your unique identifier numbers will be used instead of your names to address you throughout this discussion/interview.**

**Thank you very much.**

**Participants sociodemographic**

1. What is your full name?
2. What is your current job title?
3. What is your highest level of education, and what field did you specialise in?
4. How many years have you been working in this role?
5. Could you briefly describe your primary responsibilities and duties in your role?
6. What are some of the challenges you have encountered in your role?
7. Have you received any specific training related to vaccine safety in your current role?

**Knowledge, perceptions, and attitudes on Immunizations**

1. What vaccines are given to pregnant women? (Explore each of the vaccines given, why they are given, at what point they are provided, and where they receive the vaccines. Who gives the vaccines?)

2. Why do you think it is essential for pregnant women to receive these vaccines? (explore the COVID-19 vaccine)

3. Are pregnant women given information about the vaccines they receive? Who provides the information (personnel, source of data), what kind of information (Content), where are they given (location of information dissemination), and when are they given (Timing)? What challenges are involved in vaccine information delivery?

4. What are some cultural beliefs about vaccines in the community (explore positive and negative opinions, myths and misconceptions)? (explain) (explore COVID).

5. In your opinion, what do you think motivates pregnant women to receive or not receive vaccines? (Explore access, distance, incurred cost, the attitude of health care workers, documentation, waiting time, etc

6. How would you describe the relationship between pregnant women and health workers in the immunisation/ANC clinic? (Explore positive and negative). How do they perceive the Ministry of Health as a whole?

**Factors that influence acceptance and access to COVID-19 vaccines in pregnancy**

1. What are your thoughts about pregnant women receiving COVID-19 vaccines? Do you consider the COVID-19 vaccines safe in pregnancy? (If yes, why or why not?) (In what way do you think the introduction of COVID-19 vaccines has influenced or affected the lives of pregnant women and their unborn babies?)

2. At the introduction of the COVID-19 vaccine, pregnant women were not receiving the vaccines; at what point did the Ministry of Health decide to include pregnant women in the roll-out program? (What informed this decision?)

3. Do you think pregnant women should have been included in studies during the development of the COVID-19 vaccine? (explore vaccine trials)

4. Did pregnant women report any form of adverse event following vaccination? (Explore. If yes, what vaccine was it? What reaction(s)? Do you think the vaccine caused it or something else? How was the situation managed?

5. Do you think the information available in the community is enough for patients to decide? Do you think the health sector could do better with vaccine-related communication and education regarding the COVID-19 vaccine?

**Decision-making process**

1. Is there training or guidance provided to healthcare workers to address concerns about COVID-19 vaccines among pregnant women? (Is there a protocol to follow? How do you transfer this knowledge to other members of your team? Are there things you can do better?)

2. From our study, we observed a low uptake of the COVID-19 vaccine among pregnant women. What could be responsible for this low uptake among pregnant women? (Explore timelines, COVID and post covid period)

3. In the case of a pandemic requiring the emergency introduction of a vaccine in the future, what do you think will be the potential barriers and enablers to accept it? Learning from the experiences of the COVID-19 vaccine, what would you suggest could be done to improve service delivery?

4. From our discussions with some pregnant women, they complained about inadequate vaccine information in making decisions on vaccine acceptance. How do you think you can adequately inform them about new vaccines?

5. What advice do you have for the government/health sector in ensuring trust in the vaccine service delivery?

We have concluded the discussion. Do you have any questions or further suggestions on this subject?
