# Supplementary material for: How were pregnant women included in emergency COVID-19 vaccine delivery in The Gambia? A qualitative study of perspectives of healthcare workers and policy influencers
Source: BMJ Open. 2026 Aug 20;16(8):e118873. doi: 10.1136/bmjopen-2026-118873 (PMC13504901; doi:10.1136/bmjopen-2026-118873)
Supplement: Supplementary file 3 [file bmjopen-16-8-s002.docx]

**Workshop discussion guide: Exploring the perspectives of healthcare workers and policymakers on the introduction of new vaccines for pregnant women in the Gambia.**

**Workshop objectives**

This workshop aims to:

1. Explore the perspectives of healthcare workers and policymakers on integrating new vaccines into maternal health services in The Gambia.
2. Identify challenges, opportunities, and actionable recommendations for improving access and acceptance of maternal vaccines.
3. Provide insights to inform policy and program development for vaccine uptake among pregnant women.

Breakfast: 9:00 am – 9:30 am

Registration and consenting: 9:30 am-10:00 am

**10:00 am – 10:30 am Session 1: Introduction and setting the context (30 minutes)**

1. Welcome and workshop objectives (5 minutes)	-	Tosin

2. Overview of the VacSafe project in The Gambia (15 minutes)	-	Tosin

3. Setting Expectations (10 minutes)	-	Alpha

**10:30 am – 12:30 am Session 2: Group Discussions (120 minutes)**

**Theme 1: Healthcare Workers' Role in Vaccine Acceptance (30 minutes)**

1. Experience Sharing

a) Can you describe the role you played in promoting COVID-19 vaccines among pregnant women?
b) What successes and challenges did you encounter in this role?

2. Influencing Factors

a) From your experience, what factors do you think influence a pregnant woman’s decision to accept or decline a vaccine?
b) How do your interactions with them affect their decisions?

3. Training and Support

a) What training or resources have you received for supporting vaccine uptake?
b) What additional support or information would make you more effective in promoting vaccine uptake?

4. Recommendations

a) How can the role of healthcare workers be strengthened to improve vaccine acceptance among pregnant women?

**Theme 2: Addressing Resource Constraints and Systemic Challenges (45 minutes)**

1. Current Challenges

a) In general, what resource constraints (like staffing or medical supplies) have impacted vaccine delivery services?
b) In what ways do these constraints affect vaccine service delivery to pregnant women?

2. Systemic Issues

a) What broader challenges within the healthcare system (infrastructure, logistics, etc.) may limit your ability to integrate new vaccines if a new vaccine is introduced?
b) How do these issues affect both healthcare workers and pregnant women?

3. Potential Solutions

a) What do you think are the solutions to the problems?
   i. Challenges in resources
   ii. The health care system (policies, administration, etc.)
b) What changes within the healthcare system could improve the introduction of new vaccines specifically?

**Theme 3: Overcoming Cultural Beliefs, Misinformation, and Communication Barriers (45 minutes)**

1. Cultural and Social Context

a) In your opinion, what cultural beliefs or norms have influenced vaccine acceptance among pregnant women?
b) How do these beliefs shape vaccine uptake in the country?

2. Misinformation

a) What types of misinformation have you heard concerning COVID-19 vaccines in pregnancy?
b) In what ways has misinformation affected vaccine uptake among pregnant women in recent times (e.g. genetics, infertility, etc)?
c) In what way do you think this might affect the introduction of a new vaccine in pregnancy?

3. Communication Strategies

a) What communication strategies have been adopted within healthcare services to educate pregnant women about vaccines?
b) In your opinion, how effective are these strategies in overcoming misinformation?
c) What additional approaches would help better address barriers?

4. Community Engagement

a) How can healthcare workers and stakeholders engage more effectively with communities to build vaccine trust?
b) What role can cultural and religious leaders play in promoting the dissemination of accurate information?

**12:30 pm – 1:30 pm - Session 3: Synthesis and Actionable Recommendations (60 minutes)**

1. Group Presentations – 10 minutes each (30 minutes)

a) Each group presents their key insights and suggestions from the discussions.
b) Open the floor for questions and clarifications.

2. Developing Actionable Recommendations (30 minutes)

a) Facilitate a full-group discussion to prioritise recommendations based on their feasibility and impact.
b) Agree on practical steps to support maternal vaccine programs in the Gambian healthcare system.

1:30 pm – 2:30 pm Lunch break

**2:30 pm – 3:00 pm Session 4: Conclusion and Next Steps (30 minutes)**

1. Summary of Key Findings and Recommendations (20 minutes)

a) Recap the main points and recommendations from each group.

**2. Feedback and Closing Remarks (10 minutes)**

a) Gather participant feedback
b) Provide closing remarks, thank participants, and outline the next steps.
